# Working and health conditions in teleworkers in mandatory social isolation due to COVID-19: exploratory survey in Lima and Callao

**DOI:** 10.47626/1679-4435-2023-1139

**Published:** 2024-11-14

**Authors:** Kevin Jesus Mayma-Aguirre, Miguel Angel Burgos-Flores, Iselle Lynn Sabastizagal-Vela, Jonh Maximiliano Astete-Cornejo

**Affiliations:** Facultad de Medicina, Universidad Peruana Cayetano Heredia, Lima, Perú

**Keywords:** teleworking, occupational health, working conditions, occupational risks, COVID-19, teletrabajo, salud laboral, condiciones de trabajo, riesgos laborales, COVID-19

## Abstract

**Introduction:**

The socioeconomic and health situation of people have been affected during the COVID-19 pandemic; in Peru, preventive labor measures were taken to promote home working, which generated working conditions requiring special attention.

**Objectives:**

To describe the results of an online survey on working and health conditions in workers from Lima and Callao who worked from home during the first period of mandatory social isolation due to COVID-19.

**Methods:**

In this exploratory study, an online survey adapted from the Questionnaire on Working Conditions, Employment and Health in Latin America and the Caribbean was applied between March 21 and April 11, 2020.

**Results:**

Of the 179 workers included, 50.8% reported doing remote work, and 49.2% teleworking; 3.9% did not have necessary computer equipment for their work activities, 54.2% required more time to finish their scheduled activities and 31.8% of workers did not have an adequate environment for home working. Regarding health conditions, more than 40% reported spinal discomfort, headache, and anxiety symptoms.

**Conclusions:**

Latent problems were identified regarding the working and health conditions of the Peruvian population working from, which may represent a burden on public health not considered in the prevention measures during the pandemic.

## Abstract (es)

**Introducción:**

La situación socioeconómica y de salud de las personas se ha visto afectada durante la pandemia de COVID-19; en el Perú, y se tomaron medidas preventivas laborales que impulsaron el *home office*, las cuales generaron condiciones de trabajo de especial atención.

**Objetivos:**

Describir los resultados de una encuesta virtual sobre condiciones de trabajo y salud en trabajadores de Lima y Callao que realizaron *home office* durante el primer periodo de aislamiento social obligatorio por COVID-19.

**Métodos:**

En este estudio exploratorio, se aplicó una encuesta virtual adaptada del Cuestionario de Condiciones de Trabajo, Empleo y Salud en América Latina y el Caribe, entre el 21 de marzo al 11 de abril de 2020.

**Resultados:**

De los 179 trabajadores incluidos, el 50,8% reportaron realizar trabajo remoto, y el 49,2% teletrabajo; el 3,9% de trabajadores no contaban con equipos informáticos necesarios para sus actividades laborales; el 54,2% requirió más tiempo para terminar sus actividades programadas; y el 31,8% de los trabajadores no contaba con un ambiente adecuado para realizar *home office*. Respecto a las condiciones de salud, más del 40% refirieron molestias en columna, dolor de cabeza y síntomas de ansiedad.

**Conclusiones:**

Se identificaron problemas latentes respecto a las condiciones de trabajo y salud de la población peruana en *home office*, que pueden representar una carga a la salud pública no contemplada en las medidas de prevención durante la pandemia.

## INTRODUCTION

The population’s economic, social, and health conditions have been affected by the infection caused by the severe acute respiratory syndrome virus (SARS-CoV-2), whose disease was named coronavirus disease 2019 (COVID-19) and declared a pandemic in March 2020 by the World Health Organization (WHO).^1,2^

In Peru, the first case of SARS-CoV-2 infection was reported on March 6, 2020, prompting the implementation of preventive measures to avoid an outbreak that could lead to general community transmission. However, deficiencies in the national health system^3^ resulted in an increase in COVID-19 cases, leading to the establishment of mandatory social isolation nationwide as the main health measure, which brought negative social and economic consequences to the population.^4-6^

The social and economic consequences of the pandemic have been reflected in people’s working situation, with many individuals becoming part of the unemployed economically active population.^7^ From employers’ perspective, it was decided to implement work regimes to protect workers from unemployment situations while complying with health measures, so as to promote home working (remote work or telework) for non-essential activities or, given the characteristic of the job, hybrid work.^7-9^

In Peru two modalities of offsite work were delimited, with remote work being defined as the provision of services from home or places other than the workplace, provided that the nature of the work activity allows it during the context of health emergency due to COVID-19,^9^ whereas teleworking requires that workers agree to shift to this work modality, as well as payment for expenses with goods and services that could be incurred to workers.^9,10^ In light of the foregoing, hybrid work has been proposed as an alternative for implementation of remote work by alternating days of onsite attendance to the workplace, so that employees worked from home only one day of the week and performed onsite work on the other days of the working week.^11^

These work modalities, similar to all person-work interactions, generate working conditions linked to the organization, the environment, and the task itself, which may consist of risk factors and have a negative impact on workers’ health.^12^ In the case of remote work or telework, some working conditions require special attention, such as need of computers and communication devices for the development of activities, as well as lack of work-family balance and its consequences on workers’ general health status.^13^

A literature review on teleworking described its adverse effects on workers’ health: musculoskeletal problems (pain in body regions such as hand, neck, shoulder, back), psychosocial disorders (feeling of loneliness and depression), and other associated conditions (metabolic, cardiovascular and gastrointestinal diseases).^14^

The present study aimed to describe the results of an online survey on working and health conditions of Peruvian workers who performed remote work or teleworking during the first period of mandatory social isolation due to COVID-19.

In the present article, the term “home working” was used to cover both work modalities existing in Peru: teleworking and remote work.

We consider our results to be relevant for the development of documents with recommendations and guidance on occupational safety and health preventive measures related to home working in contexts of mandatory social isolation.

## METHODS

### STUDY DESIGN

This was an exploratory cross-sectional study with data obtained from an online survey that was available from March 21 to April 11, 2020 (first period of mandatory social isolation in Peru),^15^ as an early collaborative initiative among investigators. During this period, there was the first outbreak COVID-19 cases in Peru, making home working the mandatory work modality, except for activities described as essential by the Peruvian government.^15^ The present article applied the Strengthening the Reporting of Observational Studies in Epidemiology (STROBE) Statement Checklist for reporting cross-sectional studies, considering the items applicable to the described methodology.

### POPULATION

The study’s target population was workers who performed remote work or teleworking in the context of the first social isolation in Peru. Exclusion criteria were performing onsite or hybrid work activities during the study time frame, in order to select only individuals who worked exclusively from home.^15^ There was no population information about the total number of home workers during the evaluated period, due to the urgency in the implementation of home working, which exempted from the requirement to report it to the state labor authorities.

### DATA COLLECTION AND INSTRUMENT

This study used a self-administered survey created on Google Forms and distributed through social networks and groups linked to occupational health and occupational safety and health organizations. It was available for voluntary completion for a period of three weeks, and a total of 179 individuals reported to perform home working.

The instrument consisted of a survey created *ad hoc* to explore the general working and health conditions of the working population in Lima and Callao. It contained 37 items related to employment and working conditions included in the questionnaire named Working Conditions, Employment Conditions and Health in Latin America and the Caribbean (*Condiciones de Trabajo, Empleo y Salud en América Latina y el Caribe*, CTESLAC)^16^ adapted to the Peruvian population; furthermore, socio-occupational and health items related to home working were included, based on researchers’ knowledge about the Peruvian work reality.^9,10^ The questionnaire was divided into three sections: sociodemographic characteristics (12 items), working conditions (17 items), and health conditions (eight items). Most items consisted of close-ended questions, except for the last item on health conditions, which consisted of an open-ended question aimed at exploring other adverse situations that occurred while working from home.

### PROCEDURES AND DATA ANALYSIS

The database of the online survey was examined considering quality criteria such as: random review of records by two coinvestigators and data cleansing.

Statistical descriptive analyses were conducted using absolute and relative frequencies. The Pearson’s chi-square test was applied to compare health data between the sexes and according to working conditions. Confidence interval was set at 95%, and margin of error at 5%. Statistical analysis was performed using the STATA statistical software, version 15.0. There was no need for a pilot test, due to the short period of initial mandatory social isolation.

### ETHICAL CONSIDERATIONS

The survey contained an initial question about informed consent, which included information in the study, definitions of teleworking and remote work, and a request to provide informed consent for participation. Furthermore, the conducted survey did not collect any type of respondents’ personal data, i.e., the derived database only contained the study variables; additionally, the survey did not pose any risk for respondents’ physical or mental health. Therefore, it was exempted from assessment by an institutional ethics committee.

## RESULTS

### CHARACTERISTICS OF THE STUDY POPULATION

With regard to sociodemographic characteristics, 50.8% of the sample reported performing remote work, and 49.2% telework; furthermore. 94.4% were from the region of Lima, and 5.6% from the region of Callao. Moreover, 51.4% were women, most respondents were younger than 30 years (35.8%), and 50.8% reported higher education as their highest education attainment. Additionally, 70.4% of respondents worked in the private sector, and 63.1% identified their working regime as private activity (Legislative Decree No. 728), and the economic sectors with the highest proportion of workers were the production (25.7%) and health (25.1%) sectors (Table 1).

**Table 1:** Sociodemographic characteristics of home workers

	n	%
Type of home working		
Telework	88	49.2
Remote work	91	50.8
Age (years)		
20 to 30	64	35.8
31 to 40	47	26.3
41 to 50	44	24.6
51 to 60	18	10.1
Older than 60	6	3.4
Sex		
Female	92	51.4
Male	87	48.6
Education attainment		
High school education	2	1.1
Technical education	18	10.1
Higher education	91	50.8
Graduate education	68	38.0
Region		
Lima	169	94.4
Callao	10	5.6
Average monthly income (Peruvian soles)		
Less than 930	6	3.4
930 to 1,500	10	5.6
1,501 to 4,500	80	44.7
4,501 to 8,500	47	26.3
More than 8,500	36	20.1
Sector of work		
Private	126	70.4
Public	43	24.0
Independent	10	5.6
Economic sector		
Production	46	25.7
Health	45	25.1
Education	17	9.5
Transport and communication	17	9.5
Economy and finances	11	6.1
Energy and mining	9	5.0
Public sector	7	3.9
Housing, construction and sanitation	7	3.9
Foreign trade and tourism	5	2.8
Justice and human rights	4	2.2
Agriculture and irrigation	2	1.1
Environment	1	0.6
Others	8	4.5
Type of employment contract		
Reg.^*^ 728	113	63.1
CAS† Reg. 1057	23	12.8
Reg. 276	5	2.8
Outsourced or fee-for-service	24	13.4
Others	14	7.8
Number of people living in the household		
1	9	5
2	31	17.3
3	43	24
4	49	27.4
5 or more	47	26.3
Number of people who contribute economically to the household		
1	41	22.9
2	101	56.4
3	31	17.3
4	4	2.2
5	1	0.6
7	1	0.6
At least one family member went out to work during the quarantine		
No	136	76
Yes	43	24

* REG. = Employment regime.

† CAS = Administrative Services Contract (*Contrato Administrativo de Servicios*).

Finally, regarding family conformation, 26.3% reported living with five people or more, 77.1% said that more than one family member contributed economically to the household, and only 24% reported that some family member performed onsite work during the first mandatory social isolation due to COVID-19 (Table 2).

**Table 2:** Characteristics of home working conditions

	n	%
Equipment used in home working		
Telephone set	6	3.4
Data visualization screen	36	20.1
Data visualization screen and telephone set	137	76.5
Have the necessary equipment to work normally		
No	7	3.9
Yes	172	96.1
Appropriate processing (operation) speed for the equipment		
No	35	19.6
Yes	144	80.4
Appropriate internet speed (stability)		
No	56	31.3
Yes	123	68.7
Have the necessary computer programs (software) for their work		
No	22	12.3
Yes	157	87.7
Require more time to perform their work		
No	82	45.8
Yes	97	54.2
Online meetings are held normally		
No	51	28.5
Yes	128	71.5
		
There is a delay in achieving goals		
No	84	46.9
Yes	95	53.1
Received training for home working		
No	132	73.7
Yes	47	26.3
Time working from home per day (hours)		
Less than 2	5	2.8
From 2 to 4	19	10.6
From 4 to 6	56	31.3
From 6 to 8	60	33.5
More than 8	39	21.8
Received guidance on difficulties in working from home		
No	66	36.9
Yes	113	63.1
Their employer checked that their equipment is optimal for home working		
No	109	60.9
Yes	70	39.1
Their employer ascertained that their electrical systems/internet are safe and effective for home working		
No	152	84.9
Yes	27	15.1
Their employer will pay for services (energy, Internet, telephone, others) necessary for home working		
No	172	96.1
Yes	7	3.9
Have an appropriate environment for home working		
No	57	31.8
Yes	122	68.2
Family let them work from home normally		
No	31	17.3
Yes	148	82.7
Income reduction during quarantine		
No	136	76.0
Yes	43	24.0

### CHARACTERISTICS OF WORKING CONDITIONS

In relation to information and communication technologies (ICTs) necessary to work from home, 3.9% of workers reported not having the equipment necessary for the normal execution of their work activities. Furthermore, some workers reported deficiencies in the speed of computers (19.6%), internet speed (31.3%), and lack of availability of computer programs and software (12.3%). Also, 73.7% of respondents said that they did not receive training for home working (Table 2).

With regard to the execution of remote activities, 54.2% of workers reported taking longer to perform their work than when they worked onsite, 28.5% that online meetings were not held normally, and 21.8% reported working remotely more than 8 hours per day (Table 2).

In turn, 60.9% of respondents reported that their employer did not check if their equipment was optimal for remote activities; moreover, their employer did not ascertain electrical systems and internet in 84.9% of respondents, and only 3.9% of them reported that their employer will acknowledge further payments to cover the services required for home working (energy, water, internet, telephone line, among others).

Finally, 31.8% of workers did not have an appropriate environment for home working, 17.3% had difficulties in performing their work normally due to family demands, and 24.0% informed that mandatory social isolation due to the pandemic lead to an income reduction (Table 2).

### CHARACTERISTICS OF HEALTH CONDITIONS

**Figure 1:**
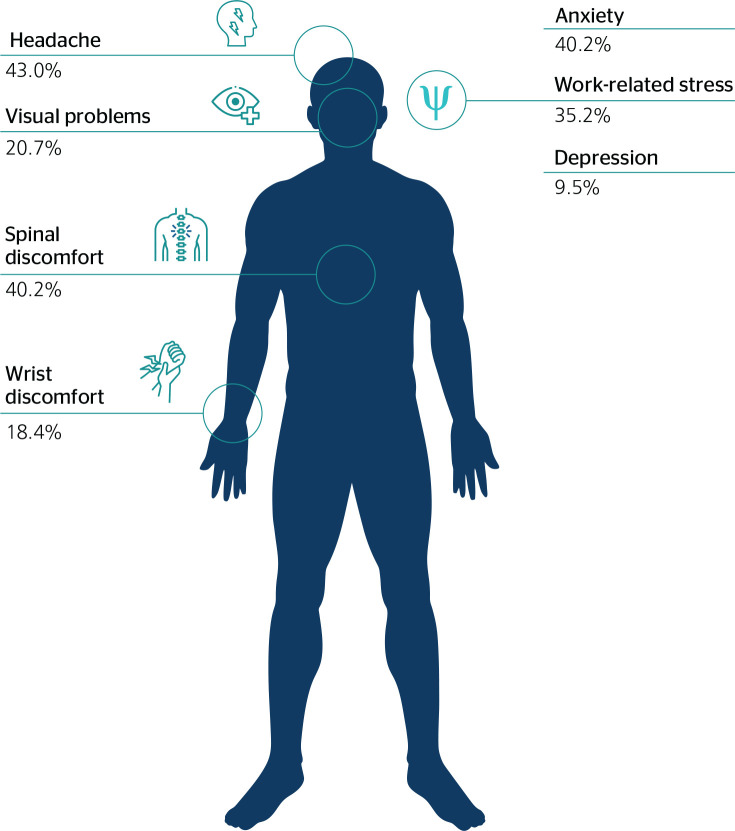
Proportion of the sample that reported health conditions while working from home.

Regarding the impact of the first days of mandatory social isolation on workers’ health, 43% of participants reported headache, 40.2% spinal discomfort, and less than one fourth vision problem and wrist discomfort. In relation to workers’ mental health, the evaluated individuals reported the beginning of anxiety symptoms (40.2%) and, in a lower proportion, work-related stress and depression (Figure 1).

Participants had the option of adding information about other situations experienced since the beginning of mandatory social isolation, and less than 4% of respondents complained of symptoms such as musculoskeletal pain at specific regions, weight gain, temperature increase, fatigue, insomnia, stomach discomfort, and respiratory problems; furthermore, they also reported prolonged boredom, fear for losing one’s job, care to older adults, and economic problems derived from the pandemic.

With regard to patients’ sex, a significant association was observed only with spinal discomfort (p = 0.002) and headache (p = 0.011), with women presenting a nearly two-fold higher incidence of these diseases.

An analysis of associations between working and health conditions revealed significant associations (p < 0.05) between at least seven working conditions and the presence of physical health problems; with regard to the presence of mental health adverse outcomes while working from home, there was a significant association with at least three health conditions (Table 3).

**Table 3:** Associations between working and health conditions while working from home^*^

	Vision problems	Writ discomfort	Spinal discomfort	Headache	Work-related stress	Depression	Anxiety
Equipment speed	0.002	0.000	0.008	0.000	0.001	0.003	0.590
Internet speed	0.031	0.001	0.005	0.004	0.002	0.000	0.014
Software functioning	0.000	0.254	0.004	0.003	0.121	0.480	0.593
Require more time to perform their work	0.010	0.006	0.015	0.570	0.001	0.154	0.006
Online meetings	0.008	0.017	0.001	0.007	0.015	0.514	0.239
Delay in achieving goals	0.006	0.830	0.017	0.031	0.000	0.042	0.017
Training in home working	0.048	0.243	0.890	0.270	0.020	0.788	0.314
Working hours per day	0.341	0.035	0.571	0.169	0.082	0.147	0.161
Receive remote support or guidance	0.095	0.258	0.850	0.039	0.369	0.360	0.438
Employer checks functioning of equipment	0.350	0.530	0.025	0.002	0.137	0.167	0.961
Employer will pay for services	0.670	0.007	0.352	0.441	0.708	0.659	0.352
Have an environment for home working	0.014	0.149	0.000	0.036	0.020	0.385	0.315
Family allowed for home working	0.001	0.029	0.001	0.144	0.910	0.970	0.680

* Values correspond to p-value of the Pearson chi-square test.

## DISCUSSION

The results of this study show that, at the beginning of the pandemic, workers and employers implemented home working to continue performing their work duties in most economic activities, a measure that was also taken by Latin American countries^17^ such as Ecuador and Chile, which were the first countries of the region to regulate teleworking in the public and private sectors.^18,19^ In Peru, with Urgency Decree No. 026-2020, remote work has been promoted since March 2020 as a measure to allow for workers to continue their work activities and to prevent spread of COVID-19. It has been shown that home working was better accepted by the private sector, consistent with reports from other countries in the region.^17^

With regard to the economic sectors, the production sector was the one with the highest proportion of homeworkers, being one of the non-essential activities that better adapted to home working; however, unlike what was reported by Delgado de la Matta,^20^ non-essential activities presented more difficulties in adapting to this work modality.

Another sector with a high proportion of homeworkers was that of health, which adapted to the remote modality in some services that could be provided virtually, in order to protect workers’ and patients’ health in the context of the COVID-19 pandemic. This same practice was implemented in care centers of different countries, such as Chile and Colombia, where guidelines were developed for the provision of telemedicine and telehealth.^21,22^

Regarding economic contribution to the household, the highest percentage of participants reported having two contributors in their household (56.4%); similar to what was reported by the 2020 national household survey (52.5%).^23^

### WORKING CONDITIONS REQUIRED FOR HOME WORKING

Among the aspects required for the implementation of home working, there are the use of any mean or mechanism that makes it possible for workers to perform their duties outside the workplace, no impact on their salary, and that the nature of the activity allows for working from home.^9^ However, our results show that 24.4% of workers had their salary reduced, contrary to the findings reported by Paladines, in which the implementation of teleworking in companies did not lead to a reduction in workers’ salaries.^17^

Although most surveyed workers had the necessary equipment for the development of their activities, more than one third had not received training or technical support in the use of technologies, which could be related to the delay in reaching goals and to extended working hours of more than 8 hours per day. In turn, a systematic review conducted by Sánchez-Toledo Ledesma that assessed studies published within a period of 20 years before the pandemic found that the most frequent negative effects of teleworking were lack of appropriate technological infrastructure, extensive working hours, and lack of organizational support,^24^ situations that persisted during the pandemic context.

Working conditions related to the household were also identified, such as the absence of appropriate work environment for home working and difficulty of workers to perform their work activities normally due to the presence of their family; these factors are related to psychosocial risk of work-family conflict.^25^ Studies have shown the relationship between home working and increase in work-family conflict, which can be explained by teleworkers’ difficulty to delimit times, spaces, and roles in the hogar^26^; these conditions may affect the quality of family relationships, work performance, and workers’ mental health, in addition to exacerbating gender disparities.^27^

### HEALTH ASPECTS TO CONSIDER WHILE WORKING FROM HOME

In relation to physical health problems, study participants reported spinal discomfort, headache, wrist joint pain, and vision problems; these findings are in line with evidence showing that poor postures, non-ergonomic equipment, and long periods of continuous teleworking lead to the development of musculoskeletal disorders in upper limbs, lower limbs and back, in addition to increased physical inactivity and sedentary behavior.^28,29^ However, this fact did not originate from the COVID-19 pandemic, since home working has been identified as a risk factor for sedentary behavior and its consequent musculoskeletal disorders.^28^

The use of visualization screens to achieve work goals became essential during the pandemic context, which can generate visual fatigue and headache,^29^ as reported in the present study. According to Tejada & Reyes,^30^ this type of discomfort may be related to inappropriate distance from the screen, difference between the lighting of the document and that of the screen, existence of reflections and glares.

With regard to mental health, the participants reported experiencing symptoms of anxiety, stress, and depression since they started working from home, findings consistent with literature reviews that describe adverse effects on workers’ health, such as the occurrence of feelings of loneliness, anxiety, stress, and depression.^29^ These symptoms are often related to excessive workload, lack of autonomy, lack of support, relational conflicts, among others,^29^ which develop in the identified associations between the working and health conditions, with slow internet and delays in achieving work goals being psychosocial risks for work-related stress, anxiety, and depression in the context of home working.

As limitations of the study, it can be mentioned that it only considered the first period of mandatory social isolation, which coincides with the first pandemic outbreak in Peru; therefore, pre-pandemic baseline data of the evaluated individuals were not available. Additionally, there was no validated instrument to assess working and health conditions adapted to characteristics of home working; hence, it was necessary to adapt an *ad hoc* survey, which will allow for obtaining exploratory results about working conditions and symptoms occurring in the context of home working, thus enabling the development of recommendation and prevention guidelines in occupational health. Furthermore, a pilot test was not conducted, due to short period of initial mandatory social isolation, which was later extended, with no knowledge on the total duration of this isolation at the beginning of the study, Finally, due to difficulties of companies to adapt to home working during the study period, a great number of workers took other measures, such as going on vacations, taking a paid leave with working hours to be compensated after the health emergency, perfect suspension of work (temporary cessation of employees and employers obligations), suspension of business activities, and dismissals; therefore, there was a great difficulty in enrolling home workers.

## CONCLUSIONS

If properly explored, the results of this study allow to identify latent problems in the implementation of home working, including lack of equipment and ICTs, reduced income, among others. Furthermore, symptoms such as headache, spinal discomfort, anxiety symptoms, and work-related stress were identified in more than one third of the surveyed sample early at the beginning of home working, which coincides with evidence reported during the pandemic and may represent a burden on public health systems, since measures to prevent, identify, and control the effects of home working during the pandemic have not been considered.
